# Æsculapian Medical Society

**Published:** 1854-12

**Authors:** T. D. Washburn, F. R. Payne

**Affiliations:** President; Secretary


### MEDICAL NEWS.

*Æsculapian Medical Society.*

The annual meeting of the Æsculapian Medical Society was held in the Baptist Church in Paris, Ills.. Oct., 18th, 1854: the President, Dr. CHARLES JOHNSON, in the chair. One of the censors being absent, Dr. S. York was nominated and elected to fill his place.

The committee appointed to revise the Constitution not being prepared to report, were continued until next meeting.

On motion the following gentleman were respectively proposed as permanent members of the Society: Drs. D. W. Stormont, James M. Steele, Charles A. Hunt, B. B. Everett, T. M. Smith, J. S. Richmond, each of whom, after being examined and recommended by the censors, was by a unanimous vote received.

On motion of Dr. Logan, the Society proceeded to the election of officers for the following year, when the following gentlemen were elected: Dr. Thos. D. Washburn, President; Dr. S. York and W. B. Duffield, V. Presidents; Dr. F. R. Payne, Secr'y; Dr. C. L. Duncan, Treas'r; Drs. Chas. Johnson, S. York and F. R. Payne, censors.

On motion, the Society adjourned to meet at 7½ o'clock P. M.

In the evening the Society met and the newly elected President was conducted to the chair. According to previous notice a respectable number of ladies and gentlemen had assembled to hear a public addess. The President introduced to the audience, Dr. S. York, who spoke in a very able and interesting manner upon the causes and nature of Quackery. After the address was delivered the audience retired.

On motion, the Secretary read a case of Strangulated Hernia, reported by Dr. S. Thompson, which was treated successfully with the knife. Also, an interesting letter addressed to the members of the Society, written by Dr. S. Thompson.

The Society then adjourned to meet at 8½ o'clock to-morrow morning.

MORNING SESSION.

On motion, the Society ordered the Treasurer to pay $16 out of the funds of the Society for the publication of Dr. F. R. Payne's address.

The reading of essays and report of cases being in order, Dr. C. A. Hunt read an able and interesting paper on "the consideration of fever in a new nosological position."

Dr. S. W. Thompson read a valuable paper on "the history of epidemic Ileo Colitis as it prevailed in Fairfield, Wayne co., Ills.," which elicited much discussion.

Dr. T. D. Washburn reported a case of Puerperal Fever."

Dr. Hamilton reported a case of complicated "Abdominal abscess."

On motion of Dr. Logan, the following resolution was unanimously adopted.

*Resolved*—That the hireling system is highly derogatory to the character of the Profession, to individual physicians, and detrimental to the best interests of society at large, and that it is incumbent upon all the members of this Society to use all honorable exertions to discountenance and discourage the longer continuance of said practice in our noble profession; further resolved, that it will be expected and required of each member of this society, who may heretofore have engaged in this practice, to discountenance it as soon as present engagements are fulfilled.

On motion of Dr. Logan it was unanimously

*Resolved*—That in the death of Jacob Lusher, this society has lost an able and efficient, honorable and worthy member; the Profession in the Wabash Valley an experienced and successful practitioner; the community a worthy and valuable citizen.

*Resolved*—That we deeply sympathize with the relations of our deceased brother.

*Resolved*—That a copy of these resolutions be furnished to the relations of the deceased by the Secretary.

On motion, resolved that Drs. Chas. Johnson, S. York and C. A. Hunt, be appointed a committe to prepare a memorial and petition to be presented to the Legislature for the suppression of Quackery, and that ey thbe requested to confer with other medical societies in this State on the subject; and further that Dr. Johnson be requested to carry up the petition and use his influence with the members of the Legislature to secure the passage of a law that will protect the public from the injury inflicted by medical pretenders.

On motion of Dr. Hamilton, resolved that the papers of this Society have accumulated in such quantity, and assumed such importance as to require a special committee to take supervision of them, said committee to be styled the 'publication committee,' and that all papers submitted to this society hereafter, shall be placed in the hands of that committee; and further, said committee be appointed by the chair. Whereupon the chair appointed Drs. C. L. Duncan, H. R. Payne and L. C. Churchill.

On motion, the Society adjourned to meet at 1½ o'clock, P.M.

EVENING SESSION.

On motion, Drs. C. Johnson, S. York and W. B. Duffield were appointed delegates to the National Medical Convention, to be held in Philadelphia next Spring.

On motion the following delegates were appointed to attend the State Medical Society—Drs. J. M. Logan, A. W. McClure and H. R. Payne.

The following gentlemen were appointed to read essays at the next meeting of this Society—Drs. A. W. McClure, H. W. Davis, H. R. Payne, C. L. Churchill, J. S. Richmond, J. D. Mitchel and D. W. Stormont.

On motion, Dr. J. S. Richmond was appointed to deliver a public address at the next meeting of the Society.

On motion of Dr. Duncan,

*Resolved*—That the thanks of this Society are hereby tendered to Dr. S. York, for his able and interesting address; and that Drs. Davis, McClure and Hunt are hereby constituted a committee to solicit a copy of the same for publication.

On motion of Dr. S. W. Thompson,

*Resolved*—That the thanks of the Society be returned to Dr. C. Johnson for his able and impartial conduct as President of this Society for the past year.

On motion of Dr. Johnson,

*Resolved*—That the thanks of the members of this Society are cordially tendered to the trustees of the Baptist Church of this place for the use of their house during the various sessions of our Society.

On motion, the Secretary was requested to furnish a copy of the proceedings of this meeting to the editors of the Paris and Marshall papers, Vincennes *Gazette*, and North-west Medical *Journal*, and that they be requested to publish the same.

The town of Marshall was selected as the most central point for holding the future meetings of the Society.

On motion, the Society adjourned to meet at Marshall on the 3d Wednesday in May next.

T. D. WASHBURN, M.D., *President*.

F. R. PAYNE, M.D., *Secretary*.

